# Trusses beyond trusses: intertwined and straw-based metamaterials and beyond

**DOI:** 10.1038/s44455-026-00027-8

**Published:** 2026-04-03

**Authors:** Elias Pescialli, Junyu Chen, Konstantinos Karapiperis, Dotan Ilssar, Dennis M. Kochmann

**Affiliations:** 1 Mechanics and Materials Laboratory, Department of Mechanical and Process Engineering, ETH Zurich, Zurich, Switzerland; 2 Data-Driven Mechanics Laboratory, School of Architecture, Civil and Environmental Engineering, EPFL, Lausanne, Switzerland

**Keywords:** Engineering, Materials science, Physics

## Abstract

Periodic beam-based metamaterials have been extensively explored for their tunable stiffness, strength, and wave dispersion. Recent efforts extend this concept by maintaining the lattice topology but replacing beams by more complex structural members—such as straws or intertwined fibers. These mechanical metamaterials leverage underexplored mechanisms from the small-scale design such as contact, friction, and sliding, or multistability and reversible reconfigurability. This provides opportunities for untapped design spaces and performance exploration.

## Introduction

Design strategies for metamaterials have traditionally mimicked nature’s building principles and structure-property relations. Examples include phononic and acoustic metamaterials inspired by atomic dispersion relations^1^, topological metamaterials mimicking the quantum Hall effect^2^, beam networks that replicate atomic lattices^3,4^ or quasicrystals^5^, periodic systems that resemble domains in ferroelectrics^6^, and cellular architectures that emulate biphasic structures emerging typically during phase separation and spinodal decomposition^7^. Fueled by new opportunities in small-scale fabrication techniques, the design and property spaces of metamaterials are continuously expanding, though most advances rely on the extension of existing design principles (e.g., leveraging periodic or random beam, plate, or shell networks) to new target functionality of interest. When focusing on (quasi-)static material properties, this has resulted in lightweight mechanical metamaterials (also referred to as *architected materials*) with, e.g., tailored stiffness^8^, strength^9^, fracture toughness^10^, wave dispersion^11^, energy absorption, and reconfigurability^12^.

In recent years, machine learning has enriched the design of metamaterials, using data-driven generative models and optimizers to identify small-scale architectures that realize target properties and functionality^13^. While powerful, most such approaches inherit two main limitations: a pre-defined design space—usually based on classical beams, plates, and shells or (low-resolution) pixel/voxel representations^7,14^—and pre-defined physics—often limited to linear or nonlinear elasticity, or taking into account some form of inelasticity, damage, contact and friction, or rate effects^15^. Topology optimization^16^ is a successful alternative technique for inverse design, yet it bears the same limitations of pre-defined design spaces and considered physical mechanisms. If we seek genuine expansions of the attainable effective metamaterial properties and functionality, those constraints must be relaxed from the outset by enriching the architectural primitives and/or the underlying physical mechanisms that the models can exploit. Nature still offers a vast library of design principles and physical mechanisms that have not been thoroughly explored in metamaterials yet promise to push the envelope of achievable form and function.

In this perspective, we illustrate the opportunity of enriching the metamaterial performance by incorporating new physical mechanisms: starting from the most common archetype of mechanical metamaterials, beam lattices, and replacing struts with more complex building blocks, specifically sliding fibers and multistable straws. This expands the metamaterial landscape accessible to both computation and fabrication. Inspired by fibrous networks and the atomic network structure of polymers, the emergent class of *intertwined metamaterials* is one such example, whose targeted exploitation of contact and friction admits reconfigurability, enhanced energy absorption, and fracture properties^17–19^. Mimicking phase transformations in solids, *straw-based metamaterials* are the second example^20^, whose myriad of stable equilibrium configurations makes them ideal for applications from impact mitigation to morphing structures^21^. Of course, both underlying physical concepts are fundamentally well understood (intertwined fibers are at the core of textiles, while bendy straws are a hit at every child’s birthday party), but using those as building blocks in three-dimensional (3D) metamaterials is outside the scope of classical design and optimization frameworks and promises underexplored property combinations and deformation-induced property tunability.

New opportunities go hand in hand with new challenges. The design of intertwined fiber networks calls for a new design framework that efficiently describes the fiber arrangement and admits its efficient optimization, from the high-level topology of fibers all the way to their geometric realization while accounting for contact and sliding. Moreover, realizing such structures requires advanced manufacturing routes that go beyond classical knitting and weaving of two-dimensional (2D) fabrics and fabricate 3D designs with fibers with and without pre-defined contact. Similarly, the design of straw-based structures needs a theoretical-computational framework to cope with the extremely high-dimensional space of equilibrium configurations and to turn the latter into actuated target reconfigurability. In addition, fabricating thin-walled straws with the required design freedom and embedding them in complex structural networks is another non-trivial challenge. But challenges are there to be tackled, and the metamaterials community has the right theoretical, computational, and experimental tools to do so.

## Intertwined metamaterials

A significant expansion of the design space of beam-based lattices is achieved by replacing the discrete strut junctions and drawing inspiration from the ancient, robust technology of textiles and woven structures^22,23^. This leads to the concept of intertwined architected materials, whose structural members are continuous, non-intersecting fibers that are interwoven or knotted into a periodic network^17–19^.

In contrast to traditional beam lattices, where joints are fixed and fracture-prone, intertwined structures deliberately harness mechanisms that are often engineered out of conventional designs: contact, friction, and sliding. These features introduce a high degree of nonlinearity and energy dissipation, allowing the material to deform significantly and resist damage through distributed load sharing^24–26^.

This behavior has a direct analog in natural and synthetic materials, particularly in the realm of polymers. In bulk polymers, large-strain deformation and toughening are governed by the sliding and entanglement of long polymer chains^27,28^. Similarly, in intertwined metamaterials, the topological entanglement between continuous strands acts as the functional equivalent of chain entanglement. The relative sliding between adjacent fibers under load allows for local stress relaxation and effective energy absorption, resulting in materials with enhanced toughness and damage tolerance (arising from the network’s ability to redistribute stress through frictional sliding and load-sharing and not in the sense of toughness in classical continuum fracture mechanics) compared to their rigidly-bonded truss counterparts.

The key to designing intertwined materials lies in exploiting a hierarchical design space that extends well beyond simply choosing a lattice topology^29,30^. Such a framework allows for the systematic creation of structures that unify seemingly disparate classes, such as woven, knotted, and closed-chain networks, exploiting control across three distinct levels:

1. Port-level topology: this is the most fundamental design choice, akin to the underlying lattice graph. It dictates the overall connectivity of strands at the unit cell boundaries (“ports”, Fig. 1a), governing the material allocation, the number of continuous fiber paths, and the resulting directional anisotropy of the effective mechanical properties.

**Fig. 1:**
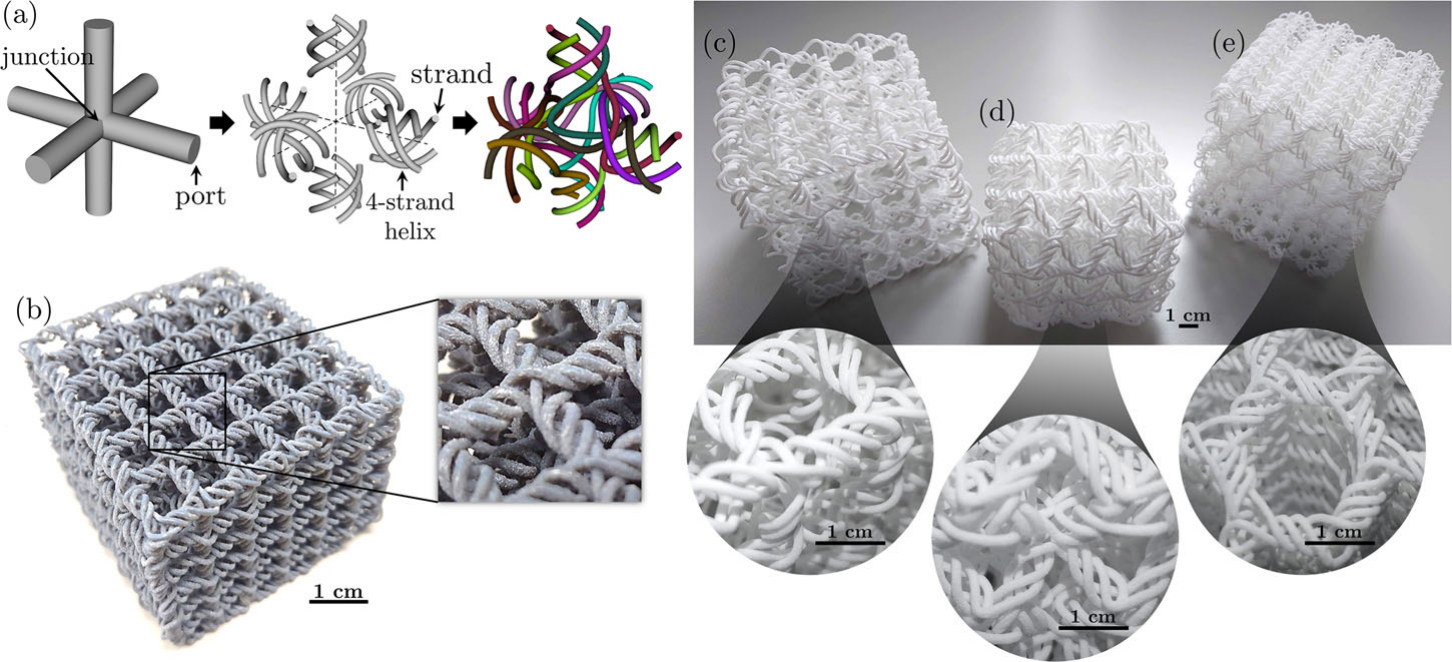
Design-to-fabrication workflow and additive manufactured intertwined architectures. **a** Design process for intertwined metamaterials (adapted from^30^), and 3D-printed samples: **b** PolyJet™ -printed cubic network (adapted from^30^); **c** selective laser sintered (SLS) cubic, **d** body-centered cubic, and **e** honeycomb-like architectures.
2. Strand-level topology and entanglement: this level resolves the precise, periodic routing of individual strands (Fig. 1a) and defines their specific topological relationship, their level of self-entanglement (knottedness) and mutual linking. This combinatorial freedom allows designers to specify an abstract entanglement function and use quantitative topological metrics (e.g., linking number) to prescribe the required complexity, which is crucial for maximizing friction-induced dissipation and toughness.
3. Geometric realization: this is the physical realization of the topology, e.g. in terms of helices routed through junctions, focusing on parameters such as strand diameter, helix radius, and junction geometry.

**Fig. 2:**
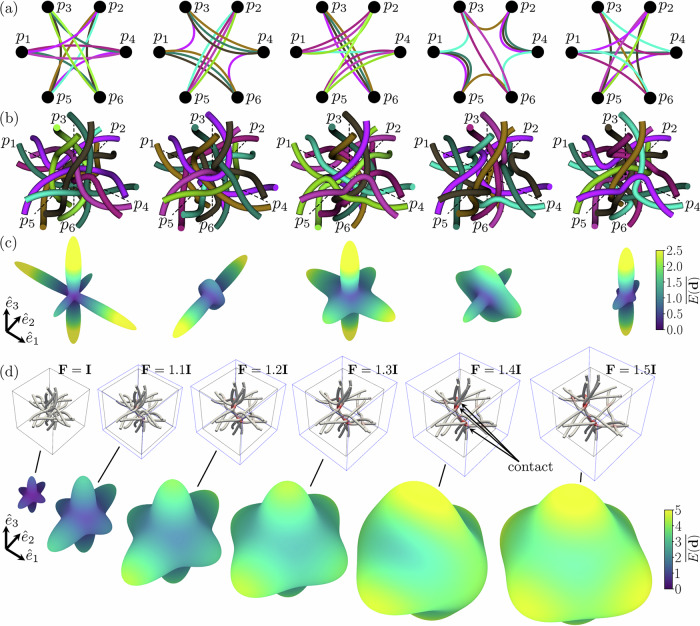
Topological design and nonlinear mechanical response of intertwined metamaterials. **a** Intertwined cubic port-level topologies, **b** fully resolved unit cells featuring the same material and geometric properties, **c** their simulated corresponding elastic initial stiffness maps, normalized with respect to the fill fraction, and **d** normalized (with respect to relative density *ρ*/*ρ*_*s*_) elastic tangent stiffness maps of a single unit cell (not included in **b**) computed at different finite (pre-)deformation gradients **F**, with contact regions highlighted in red.

By exploiting this hierarchical design, mechanical properties become tunable through independent topological variables. The port-level topology (Fig. 2a) primarily tunes the initial elastic stiffness and anisotropy (Fig. 2c). For a given port topology, the strand-level topology controls internal loop formation and promotes a variation of the fibers’ entanglement. This dictates the metamaterial’s highly nonlinear (often exponential, i.e., J-shaped^31^) mechanical response and the friction-governed behavior (toughness, energy dissipation) that emerges when inter-strand contact is engaged at large deformations, as shown in Fig. 2d. These responses were simulated via corotational beam-based nonlinear finite element analysis with a penalty-based contact to resolve discrete inter-fiber interactions at finite displacements. To quantify frictional dissipation, our model employs incremental constitutive updates for the contact law ^32^. This formulation is mathematically analogous to return-mapping algorithms in plasticity, where the Coulomb friction limit acts as a yield surface. This approach enables a rigorous calculation of the irreversible work performed during slip events, capturing the path-dependent hysteresis of the network. Because of this frictional trapping, the rest-state of these architectures is often non-unique. Unlike classical lattices, which absorb energy primarily through irreversible damage (fracture or plastic hinging), intertwined architectures utilize friction for energy dissipation that is repeatable upon resetting the network configuration. At the macroscopic scale, one can evaluate the (total) specific dissipation through the area enclosed by the force-displacement hysteresis loops (e.g., normalized by the mass) in a cyclic test. Finally, the transition from simulation to physical realization introduces further complexities. While the initial stiffness maps assume elasticity, the polymers typically used in fabrication introduce further complexities through plasticity and/or viscoelasticity. These material nonlinearities, coupled with the non-conservative nature of frictional sliding, induce strong path-dependency and history-driven effects. The shown wide mechanical variability is achieved while keeping material and geometric (helix radius and wavelength, strand radius, junction clearance) properties constant (Fig. 2b), demonstrating that these topological definitions are powerful, independent design variables.

The extraction of effective properties via homogenization of such systems remains a significant open challenge. Determining a meaningful representative volume element (RVE) is complicated by the fact that contact-induced interactions and frictional sliding can be long-range and highly history-dependent. Consequently, the convergence of effective properties may require a larger number of unit cells than traditional lattices, and the resulting constitutive models must account for the non-unique rest-states and path-dependent nature of the network.

Translating the hierarchical design of intertwined metamaterials into physical reality presents a critical, exciting challenge: realizing continuous, closely packed strands that enable crucial contact and friction without fusing, often complicated by the need to later remove intricate support material. This necessity has driven initial exploration using specialized additive manufacturing (AM) techniques like PolyJet™ (Fig. 1b), Selective Laser Sintering (SLS) (Fig. 1c–e), and two-photon lithography for micro- and nano-architectures, but the opportunities for innovative, high-fidelity fabrication are vast.

## Straw-based metamaterials

Another strategy to broaden the functionality and design space of metamaterials is the introduction of multistability. While multistability has been exploited for targeted reconfiguration in, e.g., origami/kirigami structures^33,34^, structural elements embedded with post-buckled shells and beams^35–37^, or mechanical logic^38,39^, this typically comes with specific designs and is often based on bistability to cope with the complex design space. Following the same principle as above and integrating more complex mechanisms into a classical beam-based lattice, straw-based metamaterials combine traditional lattice topologies with multistable strut replacements.

The mechanics of a single straw has a natural analogy in phase transformations of solids. Consider, e.g., the extension of a rod undergoing pseudoelasticity in shape memory alloys, which starts with a Lüders band that gradually spreads and accommodates recoverable deformation^40^. Uniaxially straining a straw in 1D achieves the same by sequentially deploying one unit cell after another in tension (and folding them in compression). Of course, reality is more complex, and each straw unit cell in 3D can accommodate many more stress-free configurations than the “simple” martensite-austenite transformation. Therefore, integrated design, modeling, and experimental approaches are required to capitalize on this type of straw-based metamaterials.

The design of straw-based metamaterials similarly benefits from a hierarchical framework—extending the design space far beyond simple lattices. This framework encompasses three separate scales that jointly enable tuning their stable configurations and energy barriers, as well as their effective mechanical properties.

1. Conical-shell design: Multistable struts in straw-based metamaterials are composed of serially connected conical shells, which serve as their fundamental building blocks (Fig. 3a). The behavior of these shells is determined by their geometry and base material, which affect the number and shapes of their stable configurations. A careful choice of parameters produces shells with one or two stable axisymmetric configurations, as well as permitting or denying bent configurations^20^.

**Fig. 3:**
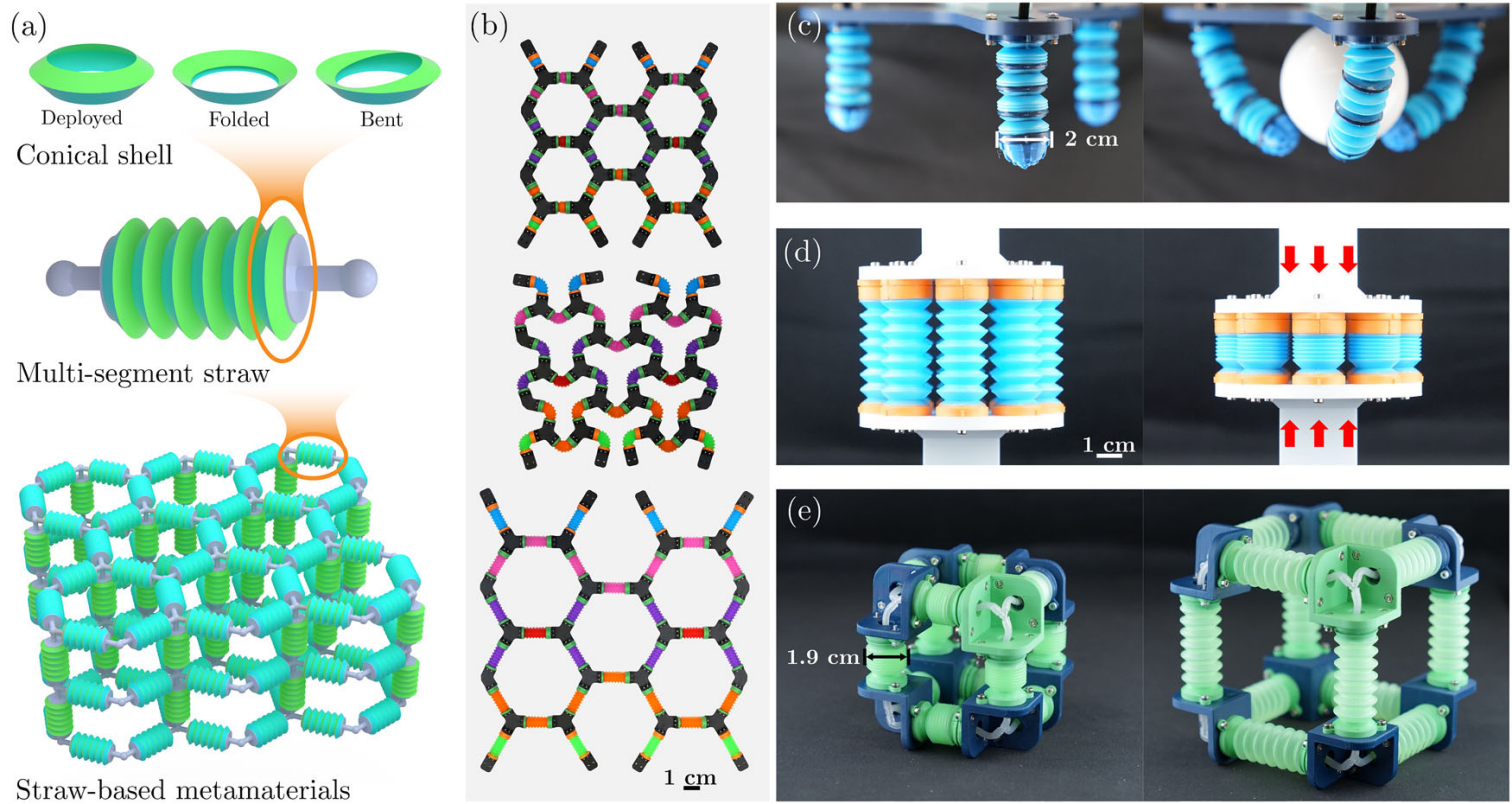
Hierarchical design and applications of straw-based metamaterials. **a** Hierarchical design---from conical shells to straws to metamaterials. **b** A 2D straw-based metamaterial in different configurations, manually deformed to demonstrate multistability and reconfigurability. Potential application of straw-based metamaterials and metastructures: **c** soft robotic gripper (directional bending enabled by eccentric string constraints), **d** energy absorber, **e** pneumatically actuated deployable structure.
2. Strut design: The serial arrangement of conical shells with different geometries (and hence different properties) results in struts with (i) a single stable configuration (behaving as bellows), (ii) multistable straws that can merely elongate and contract stably, (iii) straw configurations that can be stabilized in a myriad of multiaxial configurations^41^, or (iv) more complex struts that combine several of the above.
3. Lattice topology: Multistable struts can be assembled into different periodic or nonperiodic 2D and 3D lattices, whose effective mechanical behavior is governed by both the lattice topology and the properties of (single- or multistable) straw-like struts (Fig. 3a, b).

As in traditional beam lattices, the mechanical properties of straw-based metamaterials are determined by the interplay of their connectivity and the internal properties of their constituent struts, which in the case of straws varies with their deployment configurations (Fig. 3b). Consequentially, carefully considering all three scales (from individual conical shells to 3D structures) allows designing a hierarchical metamaterial having tunable and preprogrammed functionalities, enabling diverse applications such as soft robotic grippers (Fig. 3c), energy absorbers (Fig. 3d), and deployable structures (Fig. 3e).

**Fig. 4:**
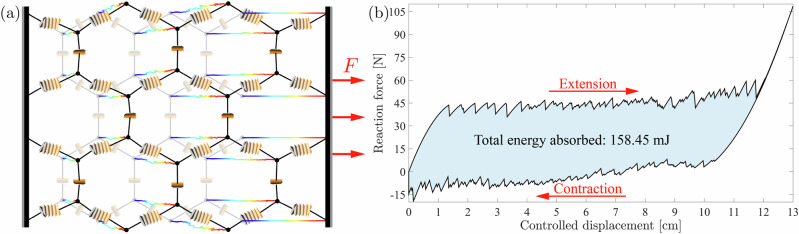
Simulated behavior of a 2D hexagonal straw-based metamaterial undergoing uniaxial displacement-controlled extension and contraction. **a** The simulated structure in its fully extended form (before the contraction phase), and in its initial configuration (opaque). **b** The force-displacement hysteresis of the actuated (rightmost) nodes of the reconfigurable hexagonal lattice.

Analogous to the hierarchical structure of straw-based metamaterials, their computational modeling approach relies on the same distinct scales. Our model utilizes a constrained formulation in which the straws are represented as serial interconnections of multistable unit cells, each governed by an analytically obtained reduced-order generalized force-displacement relation^20^. The global compatibility and interactions between the straws are enforced through holonomic constraints between nodal degrees of freedom. This framework allows linking local multistability to structure-level mechanical properties. One example is the configuration dependence of the homogenized linear elastic moduli and wave dispersion relations, which strongly depend on the unprecedentedly high number of distinct stable configurations. Moreover, the mechanical response becomes path-dependent due to snap-through transitions between configurations (Fig. 4). During loading and unloading, these instability-driven transitions generate hysteresis in the global force-displacement response, whose enclosed area quantifies the irreversible energy dissipation at the structural level. From a homogenized perspective, the effective stress-strain response resembles that of inelastic materials with apparent energy dissipation. (We note that, unlike classical plasticity—where dissipation is associated with permanent microstructural changes—the dissipation here originates from reversible transitions between stable configurations, allowing the architecture to recover its initial configuration without residual deformation or stored energy once the external load is removed.) Consequently, programmable pseudoelasticity and energy absorption can be controlled by the straw arrangement and deployment sequence, while preserving full structural recoverability.

Since the effective mechanical properties depend on the evolving configuration of many interacting unit cells, the definition of homogenized properties and an RVE becomes challenging. In the special case of strictly periodic connectivity and deformation, identical initial states, and no imperfections across all unit cells, and periodic boundary conditions, a meaningful RVE can be defined. However, outside this idealized setting, snapping and state heterogeneity lead to spatially nonuniform responses, and the concept of a unique, history-independent RVE no longer applies.

While offering broad tunability, straw-based structures are sensitive to imperfections such as manufacturing defects and geometric variations, making precise fabrication crucial. At the macroscale, the reconfigurable struts can be fabricated via blow molding, linked by additively-manufactured rigid connectors, while at the micro- to mesoscales the entire metamaterial may be produced by two-photon polymerization.

## Integration and opportunities

**Fig. 5:**
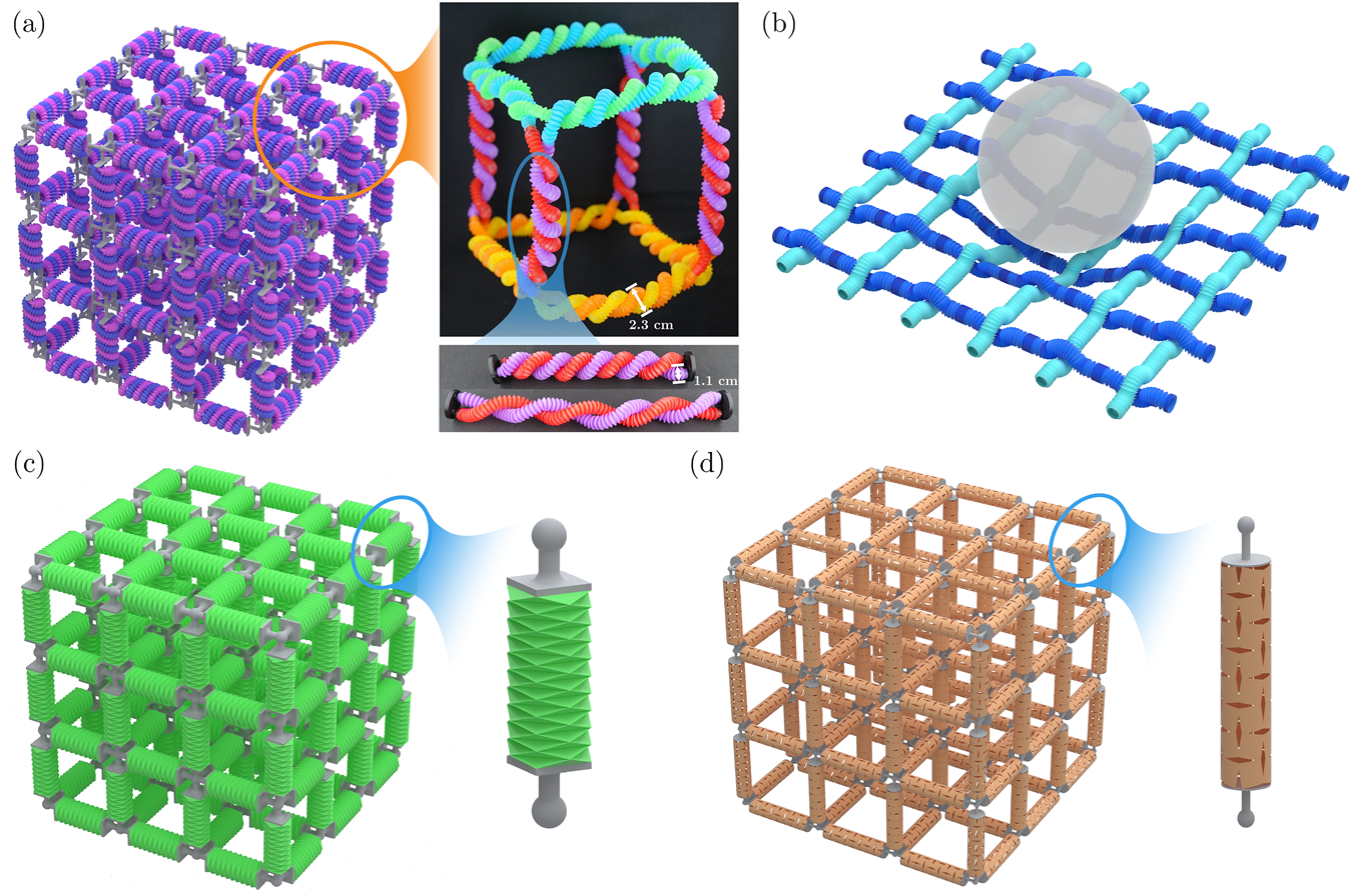
Integration of intertwined and straw-based metamaterials and future opportunities. **a** Intertwined straw-based metamaterials: a 3 × 3 × 3 lattice (left) composed of cubic unit cells (top right), each composed of intertwined straws (bottom right). **b** Woven straw network serving as a shock absorber. Metamaterials with struts replaced by other architected building blocks: **c** origami-based metamaterials, and **d** kirigami-based metamaterials.

While intertwined and straw-based architectures individually offer opportunities, the synergy of metamaterials that combine both presents another direction worthy an exploration (Fig. 5a, b). Inspired by climbing plants that grow and morph their structure while using intertwining for mechanical support, braided straws can be intertwined for increased load distribution, improving, e.g., their damage resistance. As straw elements can elongate by dozens to hundreds of percent, they can also significantly contribute to the sliding-induced friction utilized by intertwined metamaterials for energy absorption, while harnessing the internal hysteretic behavior of individual straws (Fig. 5b). Further, to limit unintentional reconfiguration of straws under weak external loads, intertwining may stabilize them and contribute to their structural integrity or activate locking mechanisms for pre-programmed motion and deformation. Such architectures may cater to diverse applications ranging from resilient metamaterials with programmable properties and efficient shock absorbers to soft robots and shape-morphing structures with rich and robust multistability.

In a broader sense, beam-based lattices can serve as the basis for a wide spectrum of metamaterials, when replacing the individual struts by other architected building blocks. Beyond intertwined fibers and multistable straws, individual struts can be realized as, e.g., thin ribbons^42^, chiral units^43^, origami or kirigami motifs (Fig. 5c, d)^44,45^, tensegrity-based components^46^, magneto-mechanical units^47^, snap-fit elements^48^, and mechanism-based modules^49^. Each class of fundamental building blocks introduces distinct deformation mechanisms, energy-dissipation pathways, stability characteristics, and functional responses, collectively enabling the design of metamaterials with programmable mechanics, rich reconfigurability, and application-specific performance.

The integration of actuation mechanisms (such as thermal^50^, optical^51^, electrical^52^, magnetic^53^, pressure^54^, and humidity^55^ stimuli) unlocks an even broader design space for the next generation of active metamaterials. Actuated fibers can dynamically modulate key properties of intertwined materials, including frictional interactions and prestress. Such control enables triggering or suppressing sliding-induced dissipation on demand, and inducing reversible tightening or loosening of braids for applications from smart wearables to diagnostics. Similarly, in the case of straw-based elements, embedding active materials can provide programmable resetting mechanisms that restore the structure after energy-absorbing events, thus enabling reuse. Their internal stability can be remotely controlled by filling the straws with fluids^54^, allowing for tuning of the energy barriers between stable configurations as well as their internal hysteresis for adaptive damping. Moreover, fluid actuation in straw-based metamaterials opens opportunities for nonlocal mechanical responses reminiscent of allosteric control in molecular biology^56^, where a stimulus applied in one location induces a controlled mechanical change at a distant site. This can be harnessed for, e.g., soft robotic gripping (Fig. 3c), strain sensing^57^, and mechanical computation^58^, as well as for protecting sensitive components by redistributing mechanical loads^59^. These are only a few examples, where the integration of actuation with intertwined and straw-based architectures transforms them from static load-bearing networks into intelligent, adaptive materials, capable of on-demand reconfiguration in real time.

## Conclusion

As this exciting new journal indicates, metamaterials have become commonplace across science and engineering. While a myriad of approaches exists to design, optimize, and fabricate metamaterials, they usually rely on modifications of a well-established design space (leveraging, e.g., beams, plates, or shells as fundamental building blocks) and on application of the latter to new effective properties (ranging from stiffness and strength to wave motion, energy absorption, and shape morphing). To break fresh grounds and steer the community in a new direction, our perspective highlights the opportunities arising from adopting the classical topological design space of beam-based lattices and enriching it by new and mechanically complex structural elements—specifically, replacing the struts in a truss by intertwined fibers or straw-like elements that engage in contact and friction (in the former case) and promote extensive multistability and hysteresis (in the latter case). This blueprint for novel types of hierarchical metamaterials not only goes beyond the classically studied design spaces, but it also promises beneficial new effective properties, made available by the emergence of new small-scale mechanisms such as frictional sliding or pre-programmed deployment. Of course, this general principle can be extended in various directions, including, e.g., active stimulus control and multiphysics settings, or integrating diverse elements (from magnets that introduce another source of multistability, through smart materials which allow for coupling with external magnetic or electric fields, to pre-explored structures such as origami or kirigami, contributing to a wider spectrum of achievable elastic properties). Challenges in theory, design, and especially fabrication present opportunities for future research.

## Data Availability

Data sets generated during the current study are available from the corresponding author on reasonable request.
